# Influence of elevation and shrub age on element accumulation in *Vaccinium myrtillus* in the alpine zone of the Low Tatras

**DOI:** 10.1007/s10661-026-15167-7

**Published:** 2026-03-07

**Authors:** Zuzana Kompišová Ballová, Michal Prodaj, Jaroslav Solár

**Affiliations:** Institute of High Mountain Biology, University of Žilina, Tatranská Javorina 7, SK-05956 Tatranská Javorina, Slovak Republic

**Keywords:** Altitudinal gradient, Element bioaccumulation, Plant age effects, Alpine shrub vegetation

## Abstract

Understanding how plant age and elevation influence elemental accumulation in alpine vegetation is essential for assessing long-term bioaccumulation processes and the impacts of environmental change in mountain ecosystems. This study aimed to evaluate the relationships between elevation, shrub age, and the concentrations of macro- and microelements in the stems of European blueberry (*Vaccinium myrtillus*) in the alpine zone of the Low Tatras (Western Carpathians, Slovakia). Stems were collected along two altitudinal transects (~ 100 m difference), and concentrations of 11 elements (S, Cl, K, Ca, Cr, Mn, Fe, Zn, Rb, Ba, Pb) were measured using energy-dispersive X-ray fluorescence (ED-XRF). Shrub age was determined from annual growth rings. Spearman’s correlation analysis revealed a significant negative relationship between shrub age and elevation (ρ = –0.62, p < 0.001), with older individuals prevailing at lower altitudes. Several elements, particularly K, Mn, and Cr, showed age-related increases in concentration, whereas multiple regression analyses identified elevation as a stronger predictor than age, with significant declines in K, Mn, and Cr at higher elevations. Principal Component Analysis (PCA) indicated that the first two components explained 55.6% of the variance, separating a general enrichment gradient (PC1) from a compositional contrast between nutrient elements and metal pollutants (PC2). ANOVA of PCA scores confirmed significant differences between transects (*p* < 0.001), reflecting altitudinal variation in elemental composition. These findings demonstrate that both biological (age-related) and environmental (elevation-driven) factors jointly shape elemental accumulation in *V. myrtillus* stems, supporting their potential use as bioindicators of long-term environmental change in alpine ecosystems.

## Introduction

Alpine environments are highly sensitive to environmental change, particularly to shifts in climate and atmospheric deposition. Mountain ranges enhance orographic precipitation and increase the deposition of airborne pollutants due to the barrier effect on air masses. Although recent reports from Central Europe (e.g., the Low Tatras) show a decline in acidic ions and heavy metals in precipitation (Sitár et al., 2016), alpine soils remain characterised by high leaching rates, low nutrient availability, and strong climatic stress, all of which influence plant growth and element cycling. At the same time, long-term warming trends—documented in high-elevation meteorological records from the Low Tatras since the 1970 s (Doležal et al., 2020)—have facilitated the upward expansion of many plant species across Europe (Steinbauer et al., 2018), altering community composition and ecosystem functioning along elevation gradients.

Within these changing alpine and subalpine environments, European blueberry (*Vaccinium myrtillus* L.) is a dominant dwarf shrub that often occupies the ecotone between dwarf pine stands and alpine meadows. It typically grows in acidic, nutrient-poor soils and forms open colonies of irregular patches shaped by soil conditions (Anadon-Rosell et al., 2016; Kandziora-Ciupa et al., 2022a). Individual rhizomes may live for several decades, although reduced rejuvenation can limit growth in older rhizomes (Nestby et al., 2011). *V. myrtillus* is generally tolerant to multiple abiotic stresses (Taulavuori et al., 2013) and may show enhanced reproductive performance at higher elevations (Pato & Obeso, 2012). Nevertheless, winter warming and reduced snow cover can increase freezing risk and shoot mortality (Bokhorst et al., 2011; Rixen et al., 2010), which the species compensates for through vigorous regrowth (Tolvanen, 1997).

The upward expansion of *V. myrtillus* has been frequently reported across European mountains (Auffret et al., 2010; Boscutti et al., 2018; Filippi et al., 2021), and is likely reinforced not only by warming but also by the abandonment of traditional grazing in the alpine zone. Although *V. myrtillus* tolerates moderate grazing (Hegland et al., 2010), its cover tends to increase when grazing ceases, as observed in formerly managed alpine meadows (Miller et al., 2010; Tasser & Tappeiner, 2002).

In addition to climatic and land-use changes, element cycling in *V. myrtillus* is shaped by both soil characteristics and atmospheric inputs. While the species is often described as relatively resistant to metal pollution (Kandziora-Ciupa et al., 2022b), this tolerance arises from physiological mechanisms such as restricted metal translocation from roots to shoots and detoxification via non-protein thiols and glutathione (Kandziora-Ciupa et al., 2013, 2017). Metal uptake and tissue concentrations are also influenced by biochemical variables (Kandziora-Ciupa et al., 2013), and rhizomes frequently contain higher elemental loads than above-ground tissues (Kandziora-Ciupa et al., 2022a; Roivainen et al., 2011; Tahkokorpi et al., 2010). Certain micronutrients, such as Mn, may accumulate strongly in *V. myrtillus* (Kandziora-Ciupa et al., 2017), largely due to the species’ occurrence on acidic soils that increase Mn mobility. Despite this, little is known about how element concentrations vary with plant age.

Age determination in dwarf shrubs through growth rings is a widely used approach for studying population dynamics and environmental influences (Boscutti et al., 2018; Casolo et al., 2020; Myers-Smith et al., 2015; Rixen et al., 2004). Applying this method to elemental analysis offers an opportunity to investigate long-term bioaccumulation processes, yet the interaction between plant age, elevation, and stem element concentrations remains insufficiently explored.

The present study examines the relationships between plant age, elevation, and element concentrations in perennial stem tissues of *V. myrtillus* in the alpine zone of the Low Tatras. Specifically, we aimed to (i) determine whether stem element concentrations reflect age-dependent bioaccumulation patterns, (ii) assess how these relationships change along an altitudinal gradient, and (iii) identify major covariation patterns among elements and their associations with age and elevation using multivariate analysis. By focusing on stem tissues, which integrate environmental exposure over many years, this study provides new insight into the temporal dimension of element accumulation in alpine ecosystems and evaluates the suitability of *V. myrtillus* as a bioindicator of element deposition along elevation gradients.

## Materials and methods

### Study area

The study area represents the alpine zone (approximately 1500–1800 m a.s.l.) in the western part of the Low Tatras National Park (Central Europe, Slovakia; 48.958964° N, 19.665914° E), established in 1978. The main ridge is built by a crystalline core; the geological subsoil in the study area consists of biotitic (oxides in % S 0.02, K 3.69, Ca 1.75, Mn 0.024, Fe 1.57) and two-mica granodiorites with pink K-feldspars (Biely et al., 1992). This bedrock produces acidic, nutrient-poor soils typical of the alpine zone on crystalline substrates. According to the Geochemical Atlas of Soils of Slovakia (Čurlík & Šefčík, 2012), ranker soils with clay–loam texture developed on crystalline substrates in the vicinity of the study area consist of K 0.78%, Ca 0.31%, Mn 0.017%, Fe 0.85%, Cr 28 mg.kg^−1^, Zn 92 mg.kg^−1^, Rb 38 mg.kg^−1^, Ba 230 mg.kg^−1^, Pb 159 mg.kg^−1^ The area belongs to a cold climatic region, with an average annual air temperature of –1.2 °C and a long-term (1951–1980) mean annual precipitation of 1142 mm (Sitár et al., 2016). Snow cover typically lasts for about 130 days per year, with a maximum average depth of 145 cm in February and March. The average annual wind speed reaches approximately 10 m.s⁻^1^ (Pobočíková et al., 2020). Observations from the nearest meteorological observation station, Chopok (2008 m a.s.l., approximately 20 km from the study area, station included in EMEP), show that the air temperature in the Low Tatras has increased significantly over the last 50 years (especially in spring, summer and autumn). This increase has been more pronounced since the 1990s. The air temperature between June and August was 2–3 °C higher between 1995 and 2015 compared to 1970–1995, with the highest temperature difference recorded in July (Doležal et al., 2020). Precipitation increased slightly and its acidity decreased during the same period, due to a decrease in primary acidifying ions such as sulphate and nitrate (Sitár et al., 2016).

### Sampling design

**Fig. 1:**
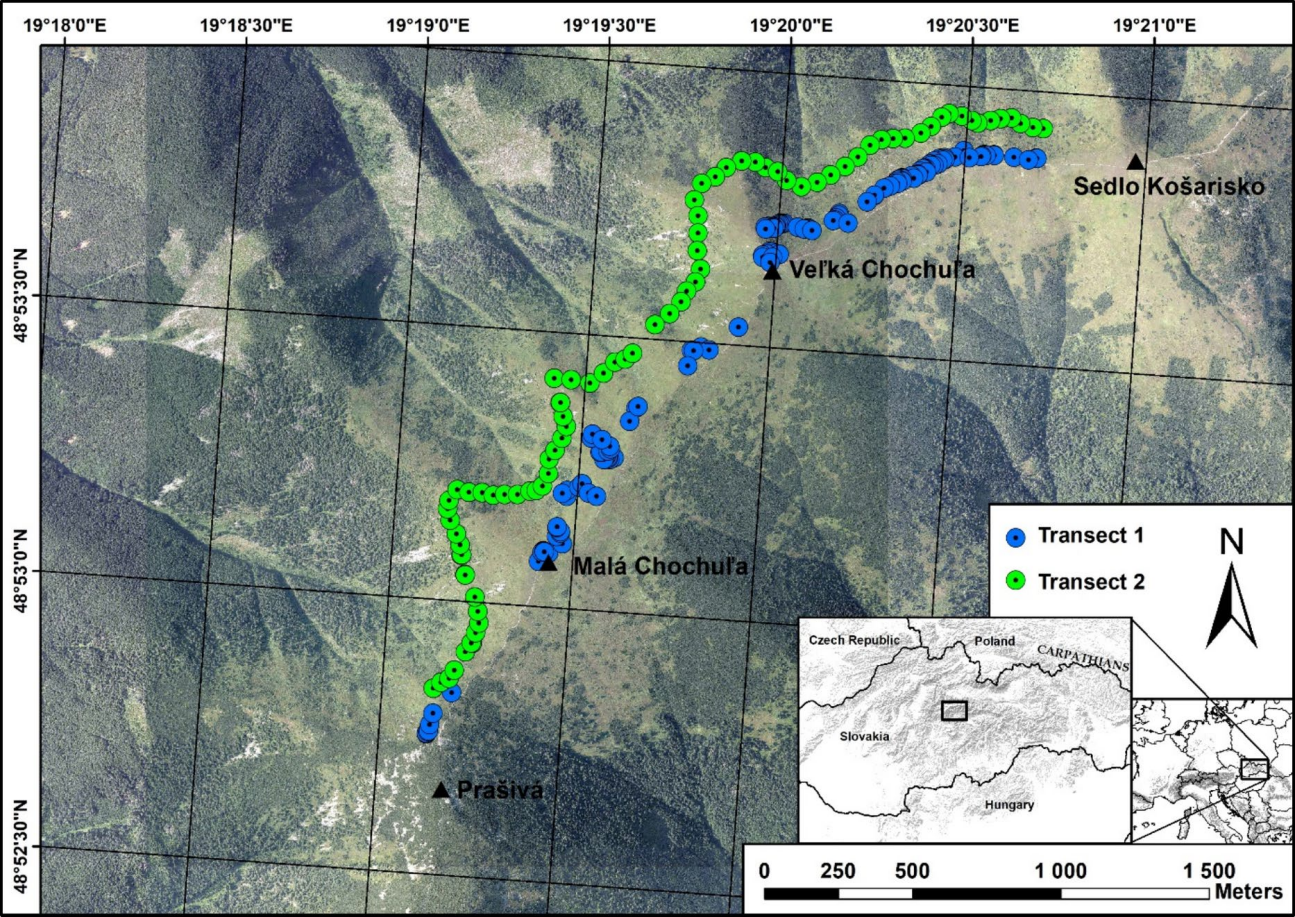
Map of the samples of European blueberry (*Vaccinium myrtillus*) collected in the Low Tatras, Slovakia (Map projection S-JTSK Krovak East North). Data source: Orthophotos from Geodetic and Cartographic Institute Bratislava (ÚGKK SR, 2022)

The sites for sampling were selected according to the following criteria: alpine zone, same geological subsoil (granodiorites), presence of European blueberry (*Vaccinium myrtillus*) on the mountain ridge, dominant abundance on downward slopes to 100 m, no grazing, and presence of abandoned marmot (*Marmota marmota latirostris*) burrows. From following criteria, we focused and investigated a suitable sites on a ridge near three hills: Prašivá (1652 m a.s.l.), Veľká Chochuľa (1753 m a.s.l.), and Malá Chochuľa (1718 m a.s.l.). Sampling was conducted in 2018 and 2019 along two altitudinal transects (Fig. 1) representing different parts of the alpine gradient. Transect 1 (T1) was located in the upper alpine zone (top of mountain ridge from 1630 to 1751 m a.s.l.), while Transect 2 (T2) was situated in the lower alpine zone (from 1563 to 1620 m a.s.l.) located approximately 100 altitudinal meters below the first transect. At each sampling site, a square plot (1 × 1 m) was used and select a dominant (the highest) *V. myrtillus* plant. To avoid pseudoreplication (due to clonal propagation through rhizomes), sites with shrubs were selected at least 10 m apart. For each plant, a basal stem section (approximately 1 cm above the ground) was collected for elemental and age analyses. In total, 195 V*. myrtillus* shrubs were sampled (T1: *n* = 116; T2: *n* = 79).

### Determination of shrub age

The age of each *V. myrtillus* shrub was estimated from annual growth rings following standard wood anatomical preparation procedures adapted from Prislan et al. (2014). A 1 cm basal stem segment was used, stored in a small plastic box for fixation before final microtome sectioning. The samples were processed by chemical drying twice in a bath of 70% ethanol for 120 min., twice in 90% ethanol for 90 min., once in 95% ethanol for 90 min., and finally, twice in 99.9% Ethanol for 90 min. The samples were then softened in a bath of xylene for 90 min., three times. Consequently, the samples were fixed in a bath by paraffin (60 °C) for 120 min., twice. The samples fixed in paraffin blocks were sectioned and then cut into 8 to 10 µm slices using a Leica RM2265 microtome (Leica biosystems, Germany). The sections were placed on glass microscope slides and fixed with glycerine albumin. After cutting and fixing of the sections, the slides were dried at 70 °C, and the remaining paraffin was melted. To clean the sections, we used a xylene bath twice for 15 min., followed by the four ethanol baths mentioned above for 15 min. each. The sections were stained with a solution of Safranine (1%) and Astra Blue (0.5%) in ethanol (70%) and prepared for microscopic analysis.

**Fig. 2:**
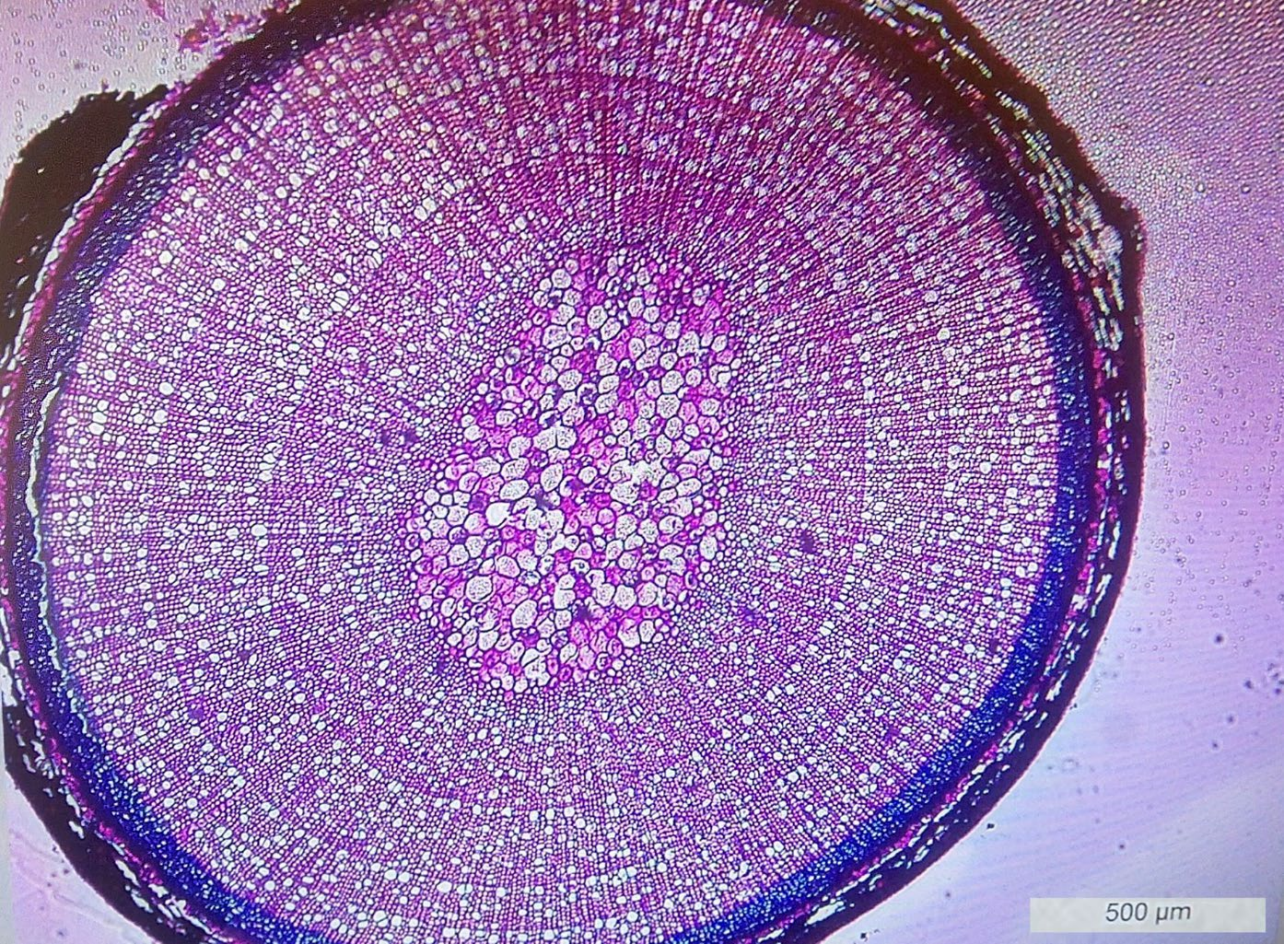
Processed and stained slice from stem of European blueberry (*Vaccinium myrtillus*) at 10 × magnification by a Leica DM6000B microscope (Leica microsystems, Germany). According to the visible growth rings, this is a three-year-old plant

Growth rings (Fig. 2) were counted under a stereomicroscope (Leica DM6000B, Leica Microsystems, Germany). Each sample was independently evaluated by two observers (in software LAS—Leica Application Suite), and any discrepancies were rechecked until consensus was reached. To verify reproducibility, a subset of 20 samples was reanalysed.

### Elemental analysis

Elemental concentrations were determined using energy-dispersive X-ray fluorescence spectrometry (ED-XRF; Olympus Vanta C Series, Olympus Corp., USA). Prior to analysis, stem samples were dried at room temperature (25 °C) and homogenised (ground to a fine powder) using a cryomill (Retsch GmbH, Germany). The samples were placed in a plastic vial of the same volume (15 mm layer).

A multi-beam measurement was used, where each measurement consisted of 3 beams for 80 s, repeated three times, and then averaged (outputs in ppm). A clean homogeneous SiO_2_ matrix without interfering elements, and certified plant reference materials (standards on a dry-mass basis) Polish Virginia Tobacco Leaves INCT-PVTL-6 (INCT, Poland) and BCR-191 (IRMM, Belgium) were used to calibrate the instrument. During measurements, instrument accuracy was verified through repeated measurements of standards. The detection limits (in ppm) were different for each element: S (100–350), Cl (40–100), K (25–80), Ca (15–45), Cr (4–10), Mn (4–10), Fe (4–15), Zn (1–3), Rb (1–2), Ba (20–25), and Pb (2–4). Analytical precision was assessed by replication of analyses when 10% of samples (randomly selected) were remeasured, and the relative standard deviations were less than 5%. The following elements were quantified: S, Cl, K, Ca, Cr, Mn, Fe, Zn, Rb, Ba, and Pb. Concentrations were expressed in mg.kg⁻^1^ dry weight.

### Statistical analyses

All statistical analyses were conducted using Statistica 8 software (TIBCO Software Inc., USA). Prior to statistical analyses, the data were examined for distributional characteristics. Variables showing pronounced right-skewness (Cr, Mn, Fe, K, Ba, and Rb) were log₁₀-transformed to improve normality and stabilize variance, while other elements were analysed in their original scale as their distributions were approximately symmetric. Shrub age (years) and elevation (m a.s.l.) were treated as continuous variables in all statistical analyses. Spearman correlation coefficients were computed among shrub age, elevation, and elemental concentrations to identify potential collinearity between predictors. Correlations were first assessed across all samples and, for comparison, within each transect.

Multiple linear regression models (GLM) were used to assess the influence of age and elevation on individual element concentrations, including interaction terms to capture combined effects. To reduce the risk of Type I error due to multiple testing, Bonferroni correction was applied to all p-values.

Principal component analysis (PCA) was performed on standardized concentrations (z-score transformed) of all elements with age and elevation included as supplementary variables. The analysis was based on the correlation matrix to ensure comparability among variables. Prior to PCA, gradient length was assessed using detrended correspondence analysis (DCA), which indicated a short gradient (< 2 SD; Axis 1 = 0.028 SD), justifying the use of linear ordination methods such as PCA (Lepš & Šmilauer, 2003). Differences in PCA scores between transects were tested using independent-samples t-tests with separate variance estimates (Welch’s t-test). Homogeneity of variances was assessed using Levene’s test. PCA scores of the first three principal components (PC1–PC3) were analysed separately.

## Results

The age of European blueberry (*Vaccinium myrtillus*) shrubs, estimated from growth-ring counts, was significantly and negatively correlated with elevation (ρ = –0.62, *p* < 0.001), indicating that older individuals were generally found at lower altitudes. Within transects, the relationship remained significant at the lower transect – T2 (ρ = –0.38, *p* < 0.001) but was weak and non-significant at the higher transect – T1 (ρ = –0.08, *p* = 0.37), reflecting a more uniform age structure in the upper part of the gradient. Since confounding of age and elevation in lower transect T2, but not in higher transect T1, we analysed correlations between elements, age, and elevation together but also separately for each transect.

Some apparent correlations (Table 1) in the total dataset (e.g., K, Cr) change direction when analysed per transect. This confirms that the overall pattern is partly due to elevation-age structure, not a true biological effect. Spearman’s correlation analysis (Table 1) revealed several moderate relationships between *V. myrtillus* age, elevation, and elemental concentrations. Across the full dataset, age showed positive correlations with potassium (ρ = 0.38, *p* < 0.001), manganese (ρ = 0.33, *p* < 0.01), and chromium (ρ = 0.25, *p* < 0.01), suggesting a tendency for these elements to increase with shrub age. However, when correlations were examined separately by transect, these relationships weakened or changed direction. In the higher transect (T1), age correlated significantly negatively with chromium (ρ = –0.26, *p* < 0.01) and showed a weak but significant positive relationship with manganese (ρ = 0.19, *p* < 0.05), whereas in the lower transect (T2) no significant age–element correlations were observed, expect Rb (ρ = −0.27, *p* < 0.05). Elevation exhibited moderate negative correlations with potassium (ρ = –0.49, *p* < 0.001), chromium (ρ = –0.40, *p* < 0.01), and manganese (ρ = –0.38, *p* < 0.01) across the whole dataset, indicating lower concentrations of these elements at higher altitudes. Within individual transects, these elevation trends were weaker and less consistent, reflecting the limited altitudinal range present within each transect. Overall, these patterns confirm that apparent element–age relationships in the pooled dataset are partly confounded by elevation, and that element concentrations in *V. myrtillus* stems are influenced by both biological (age) and environmental (elevation) factors, with site-specific variability.

**Table 1:** Spearman’s rank correlation coefficients (ρ) between shrub age, elevation, and elemental concentrations for all samples (*n* = 195) and separately for the higher (T1; *n* = 116) and lower transect (T2; *n* = 79)

Element	Age all	Age T1	Age T2	Elevation all	Elevation T1	Elevation T2
S	−0.07	−0.11	0.15	**0.17**	**0.29**	−0.08
Cl	0.01	0.06	−0.10	0.02	0.15	0.06
K	**0.38**	0.05	−0.08	**−0.49**	0.11	0.11
Ca	−0.01	−0.09	−0.04	0.01	**0.24**	0.12
Cr	**0.25**	**−0.26**	0.02	**−0.40**	**0.19**	0.10
Mn	**0.33**	**0.19**	0.17	**−0.38**	−0.16	−0.18
Fe	0.00	−0.18	−0.11	−0.06	**0.22**	0.21
Zn	−0.13	−0.17	0.04	**0.20**	**0.27**	**0.23**
Rb	0.06	0.15	**−0.27**	0.02	**0.20**	**0.42**
Ba	**0.19**	−0.02	0.14	**−0.15**	0.17	0.08
Pb	−0.02	−0.17	0.14	0.04	0.11	0.21
Descriptive statistics						
N	195	116	79	195	116	79
Mean	8.2	6.3	11.1	1640.7	1678.5	1585.2
Median	8.0	6.0	11.0	1658.0	1679.0	1585.5
Minimum	2.0	2.0	4.0	1547.6	1630.3	1547.6
Maximum	24.0	17.0	24.0	1743.0	1743.0	1607.9
Range	22.0	15.0	20.0	195.4	112.7	60.3
SD	3.87	2.47	3.75	49.70	23.02	10.92
SE	0.28	0.23	0.42	3.56	2.14	1.23

Significant correlations in bold. Descriptive statistics for age and elevation (mean, median, min, max, range, SD, SE) are provided below to supplement the sample-size information

Multiple linear regression analyses revealed that elevation had a stronger and more consistent effect than age (Table 2), showing significant negative relationships with potassium (β = –0.47, *p* < 0.001), manganese (β = –0.35, *p* < 0.01), and chromium (β = –0.32, *p* < 0.01), indicating decreasing concentrations with increasing altitude. Age, in contrast, showed weaker positive associations with several elements, including potassium (β = 0.29, *p* < 0.01) and manganese (β = 0.22, *p* < 0.05), suggesting a gradual accumulation of these elements in older shrubs. For most other elements (S, Cl, Ca, Fe, Zn, Rb, Ba, Pb), the relationships with both age and elevation were weak and statistically non-significant (*p* > 0.05). The overall explanatory power of the models was moderate (R^2^ = 0.10–0.28 for elements with significant predictors), reflecting that while both biological (age-related) and environmental (altitudinal) factors influence elemental composition, much of the variation likely arises from local microenvironmental conditions.

**Table 2:** Multiple linear regression results showing standardized coefficients (β), p-values, and model explanatory power (R^2^) for the effects of European blueberries (*Vaccinium myrtillus*) age and elevation on element concentrations, for the entire dataset and separately for Transect 1 (higher transect) and Transect 2 (lower transect)

Element	Dataset	β (Age)	p (Age)	β (Elevation)	p (Elevation)	R^2^
S	All	0.18	0.053	**0.27**	**0.003**	0.04
	T1	−0.03	0.710	**0.21**	**0.025**	0.05
	T2	0.21	0.071	−0.04	0.712	0.05
Cl	All	−0.01	0.926	−0.06	0.516	0.003
	T1	0.07	0.460	0.11	0.244	0.01
	T2	−0.08	0.484	0.07	0.575	0.01
K	All	0.02	0.796	**−0.47**	**0.000**	0.23
	T1	0.08	0.396	0.05	0.590	0.01
	T2	−0.05	0.668	0.12	0.315	0.02
Ca	All	−0.04	0.636	−0.10	0.296	0.01
	T1	−0.11	0.248	0.16	0.098	0.04
	T2	0.00	0.967	0.14	0.236	0.02
Cr	All	−0.02	0.827	**−0.48**	**0.000**	0.22
	T1	**−0.22**	**0.017**	0.16	0.094	0.09
	T2	0.08	0.503	0.17	0.165	0.03
Mn	All	0.16	0.063	**−0.22**	**0.011**	0.12
	T1	0.11	0.225	−0.17	0.071	0.05
	T2	0.16	0.170	0.00	0.995	0.03
Fe	All	−0.13	0.170	−0.16	0.087	0.02
	T1	−0.15	0.097	**0.23**	**0.013**	0.09
	T2	−0.11	0.368	0.20	0.093	0.06
Zn	All	0.01	0.932	0.09	0.317	0.01
	T1	−0.15	0.115	**0.20**	**0.029**	0.08
	T2	0.10	0.408	0.21	0.084	0.04
Rb	All	−0.09	0.308	−0.18	0.059	0.02
	T1	**0.23**	**0.014**	**0.20**	**0.035**	0.07
	T2	−0.19	0.078	**0.35**	**0.002**	0.20
Ba	All	0.17	0.070	−0.07	0.433	0.05
	T1	0.02	0.793	**0.20**	**0.034**	0.04
	T2	0.19	0.109	0.21	0.081	0.06
Pb	All	−0.02	0.824	0.06	0.547	0.01
	T1	**−0.21**	**0.030**	0.08	0.393	0.06
	T2	0.23	0.051	**0.29**	**0.012**	0.10

Significant regression coefficients (β) with *p*-values are marked in bold

Principal Component Analysis (PCA) was performed on standardized concentrations of 11 elements in *V. myrtillus* stems. The first three principal components together explained 67.8% of the total variance (Table 3). The first axis (PC1, 37.9%) exhibited strong negative loadings for most elements, particularly Ba (–0.83), Ca (–0.77), Cr (–0.77), Fe (–0.65), K (–0.63), and S (–0.61), representing a general enrichment gradient in element concentrations, where high (positive) scores indicate lower concentrations and low (negative) scores indicate higher concentrations of most elements. The second axis (PC2, 17.8%) contrasted alkali and halogen elements (K (–0.66), Rb (–0.58), and Cl (–0.56)) with Pb (+ 0.59) and Fe (+ 0.46), suggesting differences between nutrient-related and heavy metal-related compositional patterns. The third axis (PC3, 12.2%) was primarily associated with Mn (+ 0.55) and S (+ 0.40).

**Table 3:** Principal components (PC—factor coordinates of the variables, based on correlations, valid *N* = 193) indicating the mutual relationships between the content of elements in the stems of European blueberries (*Vaccinium myrtillus*) collected in the Low Tatras, Slovakia (factor coordinates greater than 0.5 or less than −0.5 in each PC column are in bold); * supplementary variables (values represents correlations of supplementary variables with PCA axes)

Element	PC1	PC2	PC3
**S**	**−0.612**	0.238	0.398
**Cl**	−0.418	**−0.557**	−0.360
**K**	**−0.631**	**−0.661**	0.090
**Ca**	**−0.774**	−0.082	0.161
**Cr**	**−0.774**	0.150	0.079
**Mn**	−0.339	−0.420	**0.546**
**Fe**	**−0.648**	0.457	−0.242
**Zn**	**−0.571**	0.210	−0.417
**Rb**	−0.373	**−0.582**	**−0.571**
**Ba**	**−0.833**	0.069	0.288
**Pb**	**−0.570**	**0.593**	−0.269
*Age	−0.157	−0.184	0.228
*Elevation	0.217	0.274	−0.154
Eigenvalue	4.16	1.96	1.34
Total Variance in %	37.86	17.79	12.18

The supplementary variables, age and elevation, were weakly correlated with the first two PCA axes. Elevation showed a positive association with PC1 (r =  + 0.22) and PC2 (r =  + 0.27), indicating that higher-elevation sites tended to have lower overall elemental concentrations but relatively higher proportions of Pb and Fe. Age was weakly negatively correlated with PC1 (r = –0.16), reflecting a tendency for older shrubs to accumulate higher amounts of elements. On the other hand, PC3 showed a weak positive relationship with age (+ 0.23). This axis likely represents internal, age-dependent variation in Mn accumulation, rather than spatial or environmental effects.

**Fig. 3:**
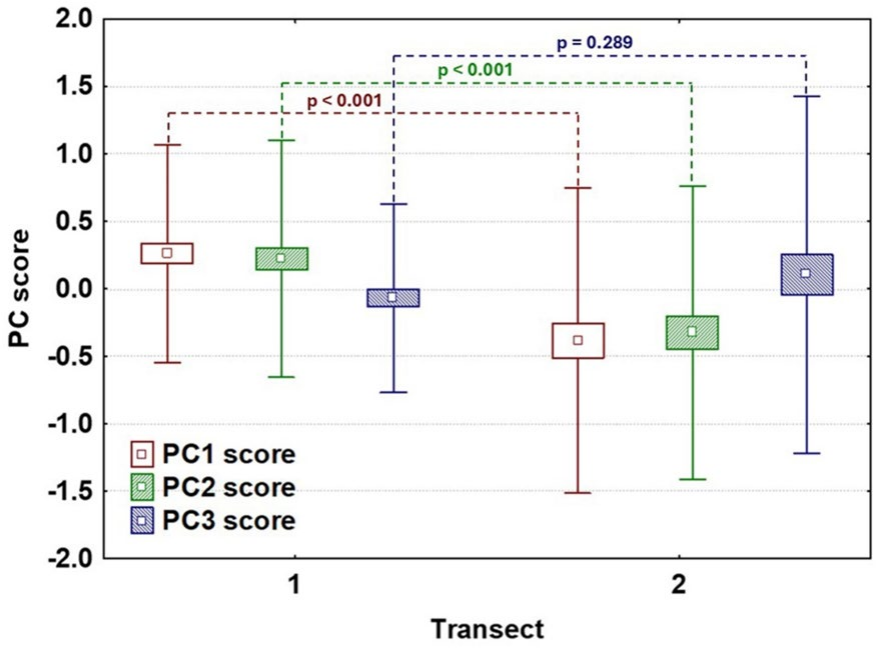
Differences in PCA scores between transects. Boxplots show the distribution of scores for the first three principal components (PC1–PC3). Significant differences were detected for PC1 (Welch’s t = 4.38, df = 130.9, *p* < 0.001) and PC2 (Welch’s t = 3.72, df = 143.5, *p* < 0.001), whereas PC3 showed no significant difference between transects (Welch’s t =  − 1.07, df = 107.7, *p* = 0.289). Boxes indicate ± standard error of the mean, whiskers represent ± standard deviation, and points denote mean values

Welch’s t-tests of PCA scores revealed significant differences between transects (T1 and T2) for the first two principal components (PC1 and PC2; Fig. 3). These differences indicate a general shift in overall element accumulation along PC1 and differences in elemental composition between transects along PC2. In contrast, PC3 scores did not differ significantly between transects (Welch’s t =  − 1.07, *p* = 0.29), supporting the interpretation that this component reflects biological accumulation processes, particularly age-related enrichment in manganese, rather than environmental variation.

## Discussion

Understanding how elevation and plant age jointly influence the accumulation of macro- and micronutrients as well as potentially toxic metals is essential for interpreting long-term environmental changes in alpine ecosystems. In this study, we analysed elemental concentrations in perennial stems of European blueberry (*Vaccinium myrtillus*) and linked them to shrub age and elevation along two altitudinal transects in the alpine zone of the Low Tatras. Several clear patterns emerged: (i) younger individuals predominated at higher elevation, confirming upward expansion of *V. myrtillus* stands; (ii) multiple elements (K, Cr, Mn, Ba) increased with shrub age, indicating long-term accumulation in perennial tissues; (iii) elevation was the stronger predictor of elemental composition, with consistently lower concentrations of several macro- and micronutrients (including K, Ca, Cr, Mn, Fe, Rb, Ba) at higher altitude; and (iv) PCA revealed distinct compositional differences between transects, reflecting both enrichment gradients and contrasts between nutrient-related and metal-related elements.

### *V. myrtillus *age structure reflects ongoing upward expansion

The age distribution of blueberries differed markedly between the two transects, with younger shrubs dominating the higher transect (T1) and older individuals prevailing at the lower transect (T2). This pattern, supported by a strong negative correlation between age and elevation (ρ = –0.62), aligns with a broader trend of shrub expansion into alpine grasslands reported from various European mountain ranges (Czortek et al., 2018; Puchałka et al., 2023; Varricchione et al., 2022; Zeidler & Banaš, 2024). Ontogenetic niche studies show that seedlings and juvenile ramets establish higher on slopes than adult plants, indicating that early life stages already occupy elevations beyond the current distribution limits (Auffret et al., 2010). Climate warming is widely considered one of the primary drivers of alpine shrubification (Rixen et al., 2010). Experimental studies confirm that *V. myrtillus* increases growth or maintains performance under elevated temperatures at higher elevations, reinforcing its capacity to colonize newly suitable habitats (Hegland & Gillespie, 2024). Observations from the Chopok meteorological station indicate that summer temperatures have increased by 2–3 °C since the 1990 s (Doležal et al., 2020), creating conditions that favour establishment of woody dwarf shrubs at higher elevations. Simultaneously, historical cessation of sheep grazing in alpine pastures may have contributed to shrub establishment, as reduced grazing pressure facilitates recolonization by competitive clonal shrubs such as *V. myrtillus* (Mrázková-Štýbnarová et al., 2025). Together, these factors support the interpretation that T1 represents an active zone of recent *V. myrtillus* expansion, while T2 reflects a more established, longer-developed *V. myrtillus* community. The age structure therefore provides a biological context for understanding spatial variation in elemental accumulation.

### Elevation as the dominant driver of elemental patterns

Elevation emerged as a stronger predictor of elemental concentrations than plant age for most elements, as demonstrated by multiple regression models and the PCA structure. Concentrations of K, Ca, Cr, Mn, Fe, Rb, and Ba were consistently lower at higher elevation, forming the basis of the dominant PCA gradient (PC1). Several environmental mechanisms may explain these patterns.

First, the study area is underlain by granodiorites and characterised by acidic, nutrient-poor soils typical of crystalline alpine substrates. In such soils, increased acidity can enhance the mobility of many elements (Bolan et al., 2003; Kumar & Radziemska, 2022), but this effect may be counteracted by strong leaching in cold, high-precipitation environments. Steep slopes and long-lasting snow cover promote downward transport of dissolved ions (Kijowska-Strugała et al., 2022), reducing nutrient availability in upper slope positions. These processes are consistent with the lower concentrations of major nutrient elements (K, Ca) and several micronutrients (Mn, Fe, Rb, Ba) observed in T1.

Second, the PCA and regression results suggest that elevation shapes not only nutrient availability but also the balance between nutrient-related and metal-related elements. PC2 showed a contrast between Pb, Fe and Zn versus K and Rb, reflecting simultaneous natural and atmospheric deposition inputs at higher elevations. High-elevation zones typically intercept air masses with greater efficiency (“mountain barrier effect”), receiving higher wet deposition of atmospheric contaminants. This may partly explain why T1 exhibited elevated Pb, Fe, and Zn despite generally lower nutrient concentrations. Overall, the strong elevation effect highlights the complexity of element cycling in alpine soils where weathering, leaching, biological uptake, and deposition act simultaneously.

### Age-dependent accumulation in perennial stems

Despite the dominant effect of elevation, shrub age also played a measurable role in shaping elemental composition. Older shrubs showed higher concentrations of K, Cr, Mn, and Ba, consistent with long-term biological accumulation in perennial woody tissues. Because stems integrate uptake over multiple years, they reflect cumulative exposure to both soil-derived nutrients and atmospheric inputs. Mn accumulation in blueberries is well documented, and our results reinforce previous observations that *V. myrtillus* can act as a Mn accumulator (Eeva et al., 2018; Kandziora-Ciupa et al., 2017). PC3 was primarily associated with Mn and showed a moderate positive correlation with age, suggesting an internal, age-related process rather than a spatial environmental pattern. Seasonal dynamics of Mn availability shaped by soil moisture (Kula et al., 2018) may further influence this accumulation.

Age also influenced microelement concentrations. Zn was moderately elevated in younger individuals at higher elevations, which may reflect physiological demands related to shoot growth and protection against freezing damage. Zn plays essential roles in enzymatic processes and stress tolerance (Kumar et al., 2021; Rehman et al., 2012), and its higher concentrations may support establishment in harsher high-altitude conditions. In contrast, older shrubs may rely more on internal recycling and storage of micronutrients in perennial tissues. These findings underscore the importance of considering plant age in biomonitoring studies, as tissue chemistry may depend not only on environmental exposure but also on the ontogeny and longevity of individuals.

An additional ecological factor that may influence elemental accumulation in *V. myrtillus* is its obligate association with ericoid mycorrhizal fungi. These fungi enhance nutrient acquisition (nitrogen, phosphorus, and micronutrients from organic substrates), particularly under acidic, nutrient-poor conditions typical of alpine soils (Cairney & Meharg, 2003; Haselwandter & Read, 1980). Although mycorrhizal colonization was not quantified in this study, variation in ERM activity along the elevational gradient could partly explain differences in elemental composition between transects. Future studies combining elemental analyses with assessments of mycorrhizal colonization and fungal community composition would help clarify the extent to which ERM symbiosis contributes to the observed age- and elevation-related patterns.

### Atmospheric deposition and metal enrichment at higher elevations

The increased concentrations of Pb, Zn, and Fe in younger shrubs at higher elevation are consistent with patterns of atmospheric deposition in high mountain environments. Even though overall deposition of acidifying pollutants has decreased in Central Europe (Sitár et al., 2016), long-range transport continues to deliver trace metals to alpine zones. Pb, in particular, remains persistent in soils and vegetation despite reductions in industrial emissions, and its mobility in acidic soils is relatively high (Brašanac-Vukanović et al., 2019). Snowpack accumulation and subsequent spring melt can further concentrate particulate-bound metals near the soil surface, increasing their bioavailability during the growing season. This likely contributes to the PCA separation of nutrient elements from heavy metals and to the observed compositional differences between transects. Thus, the combination of deposition, soil properties, and elevation-related processes explains why higher-elevation shrubs exhibited enhanced uptake of certain metals despite reduced nutrient availability.

Despite the clear patterns identified in our analyses, several limitations of this study must be acknowledged. The sampling design, based on two transects situated along a single granodioritic ridge, limits the spatial representativeness of the findings and may not fully capture the environmental heterogeneity of the alpine zone. Moreover, the absence of soil chemical measurements—particularly pH, organic matter, and bioavailable fractions of metals—precludes a direct assessment of geochemical drivers of element uptake and limits our ability to distinguish soil-derived from atmospheric sources of individual elements. Vegetation structure, including plant cover, biomass production, and litter inputs, was also not quantified; therefore, potential effects of stand productivity, nutrient cycling, and competitive interactions along the elevational gradient could not be evaluated. Finally, because samples were collected during a single time period (2018–2019) and within a relatively narrow elevational range, the results cannot be interpreted as long‑term temporal trends or as a comprehensive assessment of elevation effects. These limitations highlight the need for spatially replicated transects spanning broader elevation gradients, combined with soil chemistry, microclimatic measurements, vegetation structure assessments, and longer-term monitoring, to more fully disentangle the environmental controls on elemental accumulation in alpine *V. myrtillus* populations.

## Conclusions

This study demonstrates that both elevation and plant age strongly shape the elemental composition of European blueberry (*Vaccinium myrtillus*) stems in the alpine zone of the Low Tatras. Concentrations of several macro- and micronutrients (K, Ca, Mn, Fe, Rb, Ba) and selected metals (Cr) declined with increasing elevation, indicating reduced nutrient availability and enhanced leaching in high-altitude soils. In contrast, younger individuals at higher elevations showed elevated concentrations of Pb, Zn, and Fe, reflecting the influence of atmospheric deposition and the specific environmental conditions of exposed alpine ridges. Plant age also contributed to compositional differences, with older shrubs exhibiting higher levels of K, Mn, Cr, and Ba, indicating long-term accumulation in perennial tissues. Although age effects were evident, elevation was the dominant predictor of overall elemental patterns, as confirmed by PCA and regression analyses.

By analysing stems—tissues that integrate uptake over many years—this study provides a long-term perspective on elemental dynamics in alpine vegetation. The results highlight the importance of considering both age structure and elevation when interpreting bioaccumulation patterns. Because stems record multi-year environmental exposure, *V. myrtillus* shows strong potential as a bioindicator for monitoring long-term changes in nutrient availability, atmospheric deposition, and climate-driven shifts in alpine ecosystems.

Future research should explore the physiological and soil–plant mechanisms underlying age-dependent accumulation, and assess how ongoing climate warming and changes in deposition regimes may further influence nutrient cycling and metal uptake in high-mountain environments.

## Data Availability

The datasets generated during and/or analysed during the current study are available from the corresponding author on reasonable request.
